# Smartphone Sensor Battery Consumption: A Standardized and Reproducible Test Protocol

**DOI:** 10.3390/s26102923

**Published:** 2026-05-07

**Authors:** Florian Schweizer, Joe Yu, Elena Mille, Lara Marie Reimer, Maximilian Kapsecker, Jens Klinker, Stephan Jonas

**Affiliations:** 1 Institute for Digital Medicine, University Hospital Bonn, University of Bonn, 53127 Bonn, Germany; lara.reimer@ukbonn.de (L.M.R.); stephan.jonas@ukbonn.de (S.J.); 2 TUM School of Computation, Information, and Technology, Technical University Munich, 85748 Garching near Munich, Germany; joe.yu@tum.de (J.Y.); max.kapsecker@tum.de (M.K.);

**Keywords:** power consumption in mobile phones, sensor power consumption, smartphone sensors, iphone hardware

## Abstract

We present a low-cost, fully reproducible software and hardware protocol for smartphone sensor battery cost tests. Our pipeline combines a rigorous hardware checklist and light-sealed enclosure, a software checklist for iOS devices, and a BatteryTest app to control sensor configurations and log battery state during tests. Methodologically, we applied this standardized protocol in 30 independent analyzed test runs using six iPhone 14 Pro and three iPhone 13 Pro devices, and compared battery-life outcomes across predefined sensor conditions (idle, TrueDepth, GPS, accelerometer, pedometer, gyroscope, and rear camera), sampling rates, and sensor-specific settings. Key findings include: (i) Baseline battery life was approximately 10% higher on the 14 Pro versus the 13 Pro models under idle conditions. Sensor activation substantially reduced battery life, with GPS and camera usage exhibiting the strongest impact. (ii) Software parameters matter: the sampling rate change from 27 s to 3 s led to significantly decreased battery life in several scenarios, while reducing the location accuracy in GPS tests increased battery life by up to 20 h on the 13 Pro devices. (iii) Cross-device-generation consistency is heterogeneous. The iPhone 14 Pro lasts up to 50% longer on GPS tests, yet drains about an hour faster than the 13 Pro in camera tests. This work introduces the first standardized, and fully reproducible protocol for quantifying sensor-specific battery consumption on iPhones, enabling consistent, comparable, and low-cost energy benchmarking across device generations.

## 1. Introduction

New smartphones with updated hardware and software components are released faster than ever [1]. Apple releases several new iPhone models each fall, each including a distinct combination of sensor hardware and underlying software. For example, the company released four new models in 2025: iPhone 17, iPhone 17 Air, iPhone 17 Pro, and iPhone 17 Pro Max. The Pro models differ significantly from the base model (iPhone 15), as they include improved hardware components and therefore additional software features. Each new generation tends to have different and upgraded components compared to the previous release. These new components are usually more efficient and faster, but might also have increased battery consumption, heat generation, or computing impact [2].

iPhones typically include a variety of sensors [3], including frequently used ones like those described in this section.

- True Depth (TDS): The true depth sensor is embedded in the front of the iPhone and is mainly used for face recognition as part of iOS’ “Face ID” feature.
- GPS: GPS is used to determine a device’s current global position. Developers can set a desired accuracy for location updates received by the sensor, depending on the use case.
- Accelerometer (ACX): The accelerometer is used to measure forces acting on an iPhone through movement. This enables use cases like tracking the device’s current speed and its direction.
- Pedometer (PED): The pedometer is used by the operating system and HealthKit to passively count users’ steps throughout the day.
- Gyroscope (GYR): In addition to measuring movement through the accelerometer, developers have access to rotational forces acting on the device through the gyroscope.
- Camera (CAM): Although often not perceived as such, the camera module is also considered a sensor. Most iPhone models include either one front-facing and one back-facing camera (base models) or one front-facing and three back-facing cameras (pro models).

### 1.1. Background

#### 1.1.1. Sensor Relevance in Mobile Applications

Smartphone sensors have been used for a variety of medical applications. For example, the camera can be used to assess and monitor eye health and visual impairments [4,5,6]. It can also be used to monitor skin health [4]. As demonstrated in 2013, over ten years ago, combining smartphone cameras and neural networks is a valid option to detect skin diseases [7]. Even approaches without on-device processing can take advantage of the camera, as a 2015 review on mobile apps for melanoma detection highlights [8]. The review also highlights the importance of the iPhone ecosystem in the vast smartphone landscape, as a majority of applications included in the review were only available on that system. Cardiovascular activity, movement, and falls can be monitored through the camera and motion sensors, including the accelerometer and gyroscope [4]. Reviews in 2019 and 2021 on human activity recognition using smartphone sensors highlight the broad landscape of such applications, including the variety of sampling rates and device positions on the user’s body [9,10]. These sensors are also often used to track sleep [4], but the recent trend for consumers is shifting towards using wearables, such as the Apple Watch [11] or the Whoop [12] band, instead. Mental health and illnesses such as neurodegenerative diseases can also be assessed through an array of mobile sensors [4].

Developers use these hardware components to build various kinds of applications, but they are restricted by the operating system (iOS). iOS does not allow developers direct access to the hardware and instead provides an API to communicate with it. This means that developers do not usually have the fine-grained control that they would have on other operating systems, such as Android. The APIs represent a level of abstraction that often makes it opaque as to what is going on behind the scenes, and developers are unsure about how the sensors bring in data and how they are further processed. However, sensor APIs allow for configuration, making it increasingly complicated to judge battery impact as there is no one single implementation possibility [13].

#### 1.1.2. State of the Art in Mobile Energy Measurement

In their 2015 paper on systems to detect and tackle battery consumption issues in smartphones, Naik and Chavan list “overuse or misuse of certain types or resources” and “changing configurations to be more power-consuming” as potential reasons for increased power consumption of consumer applications [14]. These resources and configurations are likely linked to specific hardware components, which naturally use energy to function. To understand these sensor costs in detail, and to be able to make decisions about sensor usage, their power consumption must first be tested. This should happen in a standard laboratory setting, ensuring the environment is consistent and other variables are minimized. Such a protocol should also be low-cost and easy to set up, given that it might only be needed once a year when new devices are released.

Prior work on mobile energy measurement can broadly be grouped into hardware-based instrumentation, software-based or model-based profiling, and application- or sensor-level runtime studies. At the hardware end of the spectrum, Carroll et al. measured smartphone component power with external circuitry and sense resistors, enabling high-fidelity observations of individual subsystems [15]. Likewise, GreenMiner was proposed as an automated hardware-based framework for repeatable mobile-energy experiments across software revisions [16]. These approaches provide strong experimental control and precise traces, but they also require dedicated equipment, hardware access, and substantial setup effort.

Other work emphasizes software-based or model-based energy profiling. Zhang et al. proposed PowerBooter and PowerTutor to estimate smartphone power consumption online from built-in battery interfaces and learned component models, making profiling much easier to deploy in practice [17,18]. Similarly, DevScope supports non-intrusive online power analysis of smartphone hardware components, while Pathak et al.’s Eprof focuses on attributing energy consumption to application behavior and asynchronous execution patterns [19,20]. These tools improve observability for developers, but they typically rely on device-specific calibration, operating-system support, or platform assumptions that complicate direct reuse across smartphone generations.

As visible through the usage of location-based augmented reality games like “Pokémon Go” (https://pokemongo.com/, last accessed 5 February 2026) and simple location-based apps like “Maps” (https://apps.apple.com/de/app/maps/id915056765, last accessed 5 February 2026), access to sensors usually leads to increased energy demands. Ultimately, this means that sensor-dependent apps cannot be used as long as other apps that do not need to access this hardware. In their review of over 9 million user comments on apps in the Google Play marketplace for Android applications, Wilke et al. tried to understand if users actually care about the impact on battery life of an app [21]. They found that over 18% of apps had comments with complaints about energy consumption. Unsurprisingly, games exhibited the most negative comments toward battery impact, which makes sense given their computational intensity. Even well-known apps from corporate developers showed a high percentage of negative comments regarding energy usage. This research highlights that developers should care about their apps’ battery impact. First though, developers need an understanding of each component’s energy consumption with respect to their concrete usage and implementation. A study carried out with an iPhone 5S running iOS 9.0.2 with a custom SensingKit software (v0.5) already measured power consumption for various sensors (idle, accelerometer, gyroscope, magnetometer, device motion, GPS with best accuracy, microphone, and iBeacon). They found that GPS with the best location accuracy draws the most power, with the magnetometer and microphone being the most efficient aside from iBeacon tests, which are irrelevant to our work. In their setup, they used a custom “SensingKit” framework to read new sensor data as soon as they become available. The collected data were then stored locally on-device [22]. Another study by Perrucci et al. from 2011 looked at energy consumption of various smartphone sensors, including bluetooth, UMTS, and WiFi [23]. Abdesslem et al. also compared the battery impact of several mobile sensing modalities and highlighted the substantial energy cost differences between sensor choices in practical mobile sensing systems [24]. Despite detailed results regarding battery life and power draw, their paper lacks a clear protocol for hardware and software setup. The previously mentioned SensingKit study also lacks this crucial aspect that is relevant for the reproducibility of data [22]. Taken together, the literature shows that battery consumption of mobile systems can be studied with very precise hardware instrumentation, developer-oriented profiling frameworks, or application-level sensing experiments, but these strands are only loosely connected. A standardized protocol would allow more direct comparability of the data gathered by this research, leading to a broader understanding of battery impact under different sensing configurations and across device generations. In summary, existing work is limited by four recurring issues: (i) hardware-based methods are accurate but intrusive and expensive to replicate, (ii) software-based profilers often depend on device-specific models or platform support, (iii) sensor-level studies frequently use heterogeneous and insufficiently documented experimental setups, and (iv) few studies emphasize a low-cost reproducible protocol for current iPhone generations and configurable iOS sensor settings. The key research gap is therefore not a lack of evidence that sensors affect battery life, but the lack of a clear, low-cost, and reproducible protocol that can be reused to generate comparable sensor-level battery measurements across current iPhone generations and configurable iOS sensor settings while remaining practical for regular research workflows.

In their 2010 power consumption study, Carroll et al. describe a precise hardware-based setup to test the power consumption of physical smartphone components [15]. They wired in sense resistors to the relevant components inside the smartphone. While this approach is highly accurate, it is hard to replicate without the proper tools and foundational understanding of the device’s architecture.

To address this gap, we introduce a software-based sensor testing protocol that complements prior hardware-based and model-based profiling work by focusing on a reproducible application-level methodology for iPhone sensor benchmarking. The protocol is simple, cheap, and usable for different sensor categories. The battery testing is done via software measurements, but the protocol includes rigorous setup to ensure reproducibility.

### 1.2. Contributions

Based on this gap, this paper makes the following contributions:

- We present a standardized and reproducible hardware-software protocol for iPhone sensor battery benchmarking that is designed to be low-cost and practical to replicate.
- We provide and apply a dedicated BatteryTest app workflow that controls sensor configurations and systematically logs battery state under controlled conditions.
- We deliver comparative sensor-level battery-life measurements across two recent iPhone generations, including baseline and sensor-specific scenarios.
- We quantify how configurable software parameters, such as sampling rate and GPS accuracy, influence battery life under otherwise controlled conditions.
- We provide reproducible evidence that supports comparable sensor-energy benchmarking across devices and helps guide sensor configuration decisions in mobile applications.

### 1.3. Research Questions

Guided by the issues outlined in Section 1.1, we define three research questions that the new smartphone sensor battery consumption protocol should be able to answer.

**RQ1:** *What are the baseline and sensor-specific battery life times of modern iPhones when measured with a standardized hardware and software protocol?*

**RQ2:** *How do configurable software parameters (such as sampling rate and sensor accuracy) modulate that battery life?*

**RQ3:** *How consistent are sensor energy costs across device generations?*

The standardized protocol and controlled baseline/sensor test scenarios provide the basis to answer RQ1 with comparable battery-life estimates. The parameterized test design in the BatteryTest workflow (e.g., varying sampling rate and GPS accuracy) provides direct evidence for RQ2. Finally, applying the same protocol to both iPhone 13 Pro and iPhone 14 Pro devices enables a like-for-like cross-generation comparison to answer RQ3.

## 2. Methodology

We introduce the requirements for a low-cost and reproducible software and hardware protocol for smartphone sensor battery cost tests. Consequently, we present the resulting protocol on three levels: external setup, preparation pipeline, and the newly developed BatteryTest iOS application. Lastly, we go over the methodology of our first round of 30 sensor-specific battery tests.

### 2.1. Requirements

As we discussed in Section 1.1, having a rigorous protocol for both the software state and the physical hardware setup is important for the reliability and reproducibility of the data. As such, this section presents the hardware and software requirements for our proposed test protocol.

#### 2.1.1. Hardware Setup

As we are testing total battery runtime until depletion, devices must always be charged to 100% before starting a test run. We selected 100% SoC deliberately because our primary endpoint is end-to-end device runtime under standardized sensor configurations, not electrochemical characterization of the cell at a specific operating point. Starting from a common full-charge state maximizes the observable discharge window and follows established device-level battery-life benchmarking practice, where tests are recommended to begin with a fully charged battery under otherwise standardized baseline conditions [25]. We acknowledge that lower or mid-range SoC can be advantageous in studies focused on voltage-based SoC estimation, because lithium-ion open-circuit voltage depends on relaxation behavior, temperature, and aging [26,27]. However, this concern is less central in our protocol because we do not infer energy consumption from terminal voltage curves; instead, we compare total runtimes between sensor conditions from the same verified starting state. In addition to having a fully charged battery, the devices should also be removed from any cases, and charging cables should be unplugged.

As the protocol presented in this paper is designed to work for a wide range of smartphone sensors, the lab environment has to adhere to a variety of requirements. In general, external influences on the devices should remain constant in order to guarantee the independence of results across test runs concerning the environment of those runs. Light levels, intensity, and color should remain consistent across runs and devices, as iOS has functionality built into the operating system (OS) that observes ambient light and adapts the screen correspondingly. As it is unknown how exactly this is implemented on the OS-level, a constant, dark environment is required. This consistency is achieved by placing all devices in a thick wooden box that does not allow any light to hit the devices while closed. As several sensors, such as the accelerometer and gyroscope, which react to device movements, are included in the list of sensors to be tested, movement control is also a relevant variable. As such, the device should be kept stable and motionless. Placing the devices on a table could introduce disturbing movement, which should be avoided.

#### 2.1.2. Software Setup

Similar to the hardware setup, it is relevant that the state of the device’s software is constant across devices and test runs. While this cannot be fully controlled due to the private nature of the iOS operating system and system apps, which might execute work in the background even if no settings indicate so, we aim to minimize uncertainties. For example, iOS sometimes does file system work after major updates. In the context of this protocol, devices should not be updated, yet it is possible that similar tasks still run in the background, independent of updates.

The devices should have no third-party apps installed. Background activity should be minimized to ensure consistency. This includes app-level and system-level activity (such as Apple’s iCloud services, incl. Find My). The same operating system and application version should be installed on all test devices for a given test, ensuring that background activity is consistent across devices.

### 2.2. Resulting Protocol

#### 2.2.1. External Setup

**Figure 1:**
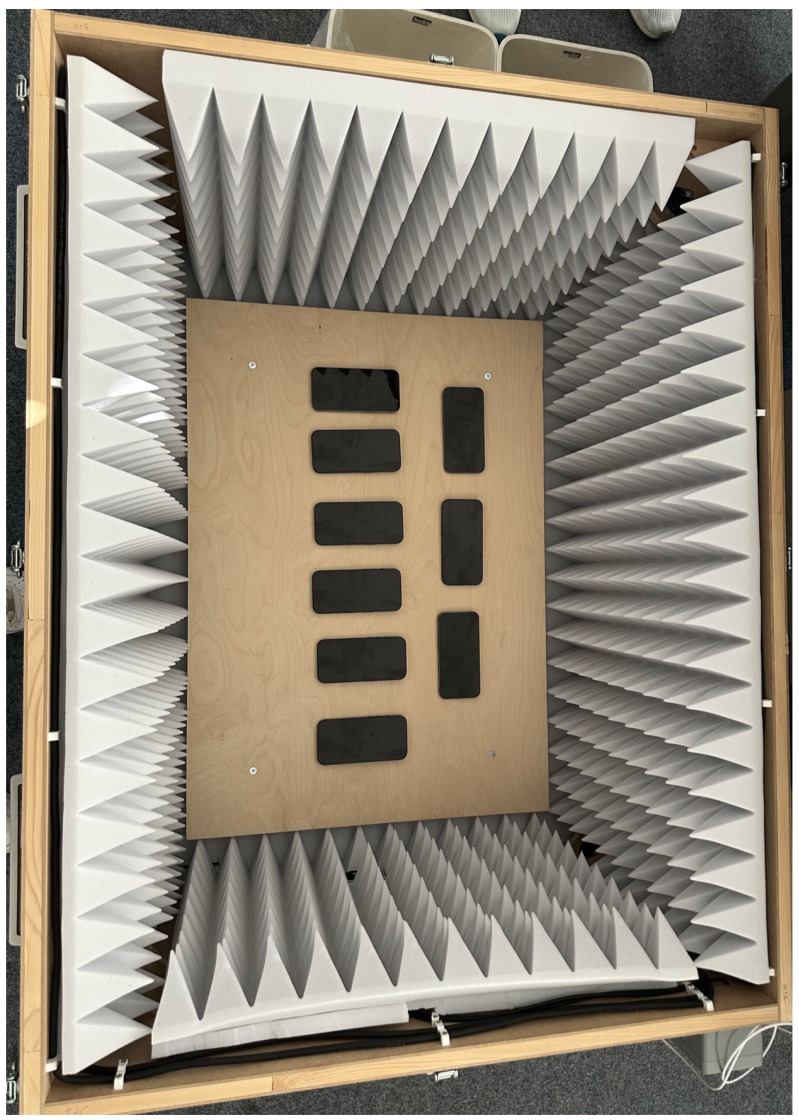
Top-down photo of the devices inside the wooden box during an idle test, illustrating the physical device arrangement used throughout the controlled indoor laboratory setup.

**Figure 2:**
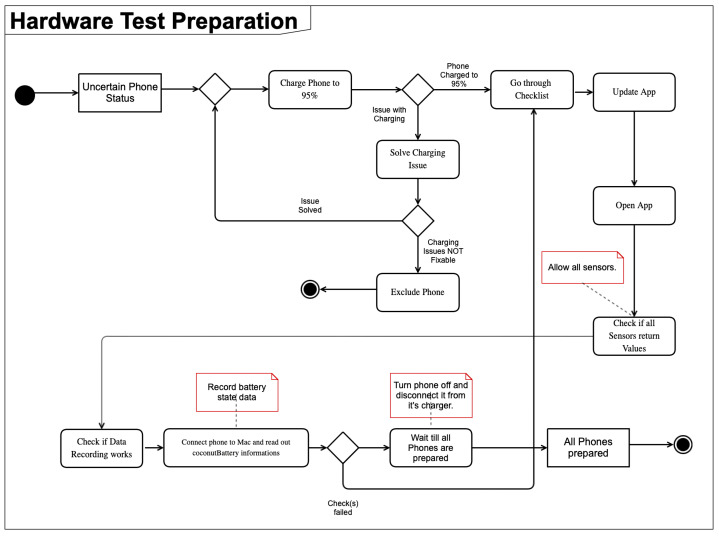
Activity diagram for the hardware test preparation.

To address several of the potential disruptors mentioned in Section 2.1, a sturdy wooden box is placed on the lab floor. Floor placement ensures stability and reduction of movement, which could impact the reliability of specific sensors, such as the accelerometer. In pilot setup checks, the box was placed indoors, outside direct sunlight, and on a location with no noticeable floor vibration. These checks were observational and not instrumented with dedicated vibration or illuminance sensors. We used a wooden enclosure intentionally, as it provides light shielding without creating a metal enclosure around the devices that could interfere with radio-based sensing scenarios. The physical arrangement inside this enclosure is illustrated by the top-down photograph in Figure 1. The broader environmental control was intentionally simple rather than chamber-based: all runs were conducted in the same office/laboratory building environment with closed windows, low foot traffic, and typically overnight execution to reduce external interference. Because this surrounding environment was an ordinary indoor workspace rather than a dedicated climatic test chamber, we did not create a separate figure for the room-level setup. Before the devices are placed in their final study position inside the box, they are all charged with a rigorous protocol, displayed in Figure 2. This charging protocol ensures a full and comparable battery state before each test.

#### 2.2.2. Preparation Pipeline

As described in Section 2.1, a rigorous process for a consistent software environment is crucial (see Figure 2). To ensure such an environment that promotes consistency across test runs and devices, a rigorous software setup pipeline is used (see Table 1). Before every run, each device is factory reset, ensuring that no third-party apps are installed or running in the background. The protocol requires verifying that each device is fully charged (100% SoC) before the run starts. In this study, we implemented this verification using both the iOS battery indicator and the third-party software coconutBattery (v3.9) [28]. Thus, coconutBattery is part of our experimental implementation, not a mandatory component of the protocol itself.

As the setup starts with an uncertain status of the phone, it is first charged to 95% capacity. As preliminary checks showed mismatches between iOS-reported and coconutBattery-reported SoC near full charge, we used coconutBattery to validate the pre-test charging state in this study. Rather than lowering the starting SoC, we therefore made the start criterion stricter and only began a run after charging had completed and the device was verified as fully charged by both indicators. If any issues arise during charging, such as the device being stuck at a certain battery percentage for a prolonged period of time, the process restarts after the device has been discharged significantly. Devices with non-fixable charging issues are excluded from the test run. For cross-generation analyses, we additionally recorded each device’s reported maximum battery capacity before runs to document battery health differences (including degradation in pre-used devices). During the test runs themselves, battery progression is tracked via software-reported state-of-charge (SoC) only. No dedicated hardware-level current or power instrumentation was used, as this would conflict with the goal of a low-cost and easily reproducible protocol. Next, the software checklist (see Table 1) is being worked through.

As we suggested earlier, the device state should be checked and any inconsistencies should be eliminated before conducting battery tests. This includes the standard approaches like turning on Airplane mode and turning off WiFi and Bluetooth. In addition to these, we intentionally extend our preparation protocol to include disabling automatic updates to promote software consistency across devices and test runs. We also monitor external factors such as device temperature before each test run and make sure that devices are not in an extreme state, as elevated and reduced temperatures can impede battery life [29,30]. Specifically, all devices were required to be below 35 °C before a run started, in line with Apple’s recommended operating conditions for stable battery performance [31]. Humidity was not instrumented in this study; instead, we reduced environmental variability by conducting all tests in the same indoor laboratory setup and enclosed box configuration.

#### 2.2.3. BatteryTest App

In addition to the preparation pipeline, a software application to enable sensor access and logging was developed. The BatteryTest application includes a framework to test various sensors regarding their battery impact. The app supports the screen (no sensors, idle test), AmbientLight, TrueDepth, Gyroscope, GPS, Accelerometer, Pedometer, and Camera. Camera and GPS can also be tested synchronously to understand the effect of multiple power-hungry sensors running at once. Once having selected a sensor to be evaluated, corresponding settings are shown. This includes screen style (black, white, red, animation), location accuracy (nearest ten meters, hundred meters, best, best for navigation), frame rate (30, 60, 120, 240 frames per second), and sampling rate (3 to 27 s, with 3 s intervals). The sampling rate refers to the interval in which battery data are read and saved. A systematic review on human activity recognition using smartphones highlighted the many different sensor configurations and sampling rates used, making this an essential part of the BatteryTest application [10]. For the controlled experiments reported here, we selected 3 s, 9 s, and 27 s as representative low, medium, and high intervals. These values form a constant factor-of-three progression, which allows sensitivity analysis across clearly separated temporal resolutions while keeping the design compact and reproducible. We do not expect the sampling rate to have a noticeable impact on battery life during the idle tests, as computational load is minimal. After configuring the test, a black screen (or colored in the case of the screen test) is shown in fullscreen mode. Using OLED-based iPhone models, this ensures minimal impact on battery life while keeping the application running in the foreground. This applies to all sensors, including camera tests: no sensor output is rendered in the UI, as the protocol is designed to isolate baseline sensor-activation costs under consistent low-overhead conditions. In the previously described interval (sampling rate), battery and sensor data are being read and saved to local storage through the CoreData framework. It is important to note that there are two data streams to ensure no loss of battery state data: The first stream records software-reported SoC at each sampling interval and saves it locally. In implementation terms, this SoC stream is based on ‘UIDevice.current.batteryLevel’, observed via ‘UIDevice.batteryLevelDidChangeNotification’. This stream is the primary source for battery-life estimation in the presented protocol and can be reproduced with the BatteryTest app alone. The second stream collects sensor data at the same configured interval, but as it accesses hardware APIs, it might not follow the nominal rate as precisely as the SoC stream. By default, the test continues running until the device turns off, which is assumed to happen when the battery runs out. Alternatively, e.g., during debugging or when a test run has to be aborted, a long press gesture anywhere on the screen also stops the test. Ongoing data are persisted in any scenario, as they are always directly saved to the file system. This means that unexpected software issues, such as crashes, will not cause a loss in data. BatteryTest is publicly available as open source software under the MIT license. The exact version used for all experiments corresponds to release v1.0-study-release and is permanently archived via Zenodo (DOI: 10.5281/zenodo.18800055) [32].

**RQ1 (baseline and sensor-specific battery life times)** is addressed by our idle test and the corresponding sensor-specific tests.

**RQ2 (configurable sensor parameters)** is addressed by the three sampling rates and parameters such as location accuracy and camera resolution.

**RQ3 (consistency across device generations)** is addressed as we included a group of iPhone 13 Pro models and a group of iPhone 14 Pro models.

### 2.3. Battery Tests

A total of 29 independent battery test runs were analyzed, categorized by their test scenario (kind of sensors used) and sampling rate (frequency of sensor usage). Each run was a separately prepared full-discharge experiment with the same cohort of nine devices measured in parallel (six iPhone 14 Pro and three iPhone 13 Pro devices). One additional attempted run had to be discarded because the box was opened and the setup was disturbed during the experiment, as discussed in Section 4.4. Table 2 summarizes the analyzed runs and their respective configurations, with 5 being idle, 6 TrueDepth, 5 GPS, 3 each of Accelerometer, Pedometer, and Gyroscope, and 5 camera-related runs (including one camera + GPS run). The number of independent repetitions per exact configuration was not uniform by design: idle 9 s was repeated three times, idle 3 s and 27 s once each; TrueDepth 9 s was repeated three times, TrueDepth 3 s twice, and TrueDepth 27 s once; GPS 100 m and best accuracy were repeated twice each, while GPS 10 m was run once; each motion-sensor sampling-rate configuration was run once; camera 30, 60, 120, and 240 fps were each run once, and camera + GPS was run once. We concentrated repetitions on baseline and high-priority configurations that support our main comparative claims and formal inferential analyses, while the singly executed configurations should be interpreted as exploratory estimates under the same controlled protocol. The full dataset generated during the experiments is publicly available via the Bonndata data repository [33] under DOI: 10.60507/FK2/LSJGWW.

In order to create a baseline of device power consumption without a specific sensor running, the idle tests were done. During these, the screen was turned black, as seen in Figure 1, and the app did no computation except for saving the state of charge (SoC) in the interval set through the sampling rate. Additionally, the rest of the supported sensors were also tested. For motion-related sensors in particular, the deliberate design goal of this protocol is to estimate baseline sensor-activation costs under controlled and reproducible laboratory conditions, not to mimic realistic in-motion usage patterns.

Our hardware setup had to be adapted slightly for the TrueDepth test, as the corresponding sensor only activates if a face is detected. While a popular approach is to place a mannequin head in front of the device, this did not fit in our wooden box. We decided to use a face printed on a piece of paper instead [34,35].

After carrying out all the individual sensor tests, we also conducted one multi-sensor test with the camera being set to 60 frames per second while the GPS sensor was also running with best accuracy. This was done to gauge the influence of one less isolated sensor on battery life while the system is under a more realistic load.

When assessing hardware components, it is crucial to write code closely to the components with as few intermediate layers as possible.

This limitation eliminates a cross-platform study design, as we would have had to use a cross-platform framework such as React Native [36] to support both iOS and Android devices. As our research group has a background in iOS development and the landscape of Android devices is much broader than that of iOS devices, we decided to test iPhones exclusively. We used 6 iPhone 14 Pro devices and 3 iPhone 13 Pro devices during our sensor tests.

### 2.4. Data Analysis

For data analysis, two separate pipelines were used to prepare and aggregate the data and later to analyze and visualize them. First, we transferred the collected battery and sensor data from each device to a laptop. The data were grouped by test run in a regular folder structure. Preliminary analysis (median run times across device families and coefficients of variation) and visualization of battery level through line graphs was done with a custom macOS application written in Swift 5. For any further analysis and visualization, we used Python3 and Jupyter Notebooks with matplotlib, pandas, and scipy.

We calculated the median run times per device group and the corresponding coefficient of variation. We visualized battery run times per sensor in box plots separated by refresh rate to give a quick visual overview of the collected data. Additionally, we created tables with an overview of the previously calculated median run times per device group. Then, we ran Holm-adjusted, paired two-tailed *t*-tests comparing battery life during idle tests with battery life during specific sensor tests per device for statistical significance. To analyze the effect of the sampling rate (3, 9, 27 s) on battery life during idle tests, we employed a one-way repeated-measures ANOVA with subject factor deviceID. Sphericity was tested with Mauchly’s test, Greenhouse–Geisser correction was applied where violated. Pairwise comparisons were Holm-adjusted and effect sizes are reported as partial eta squared (ANOVA) and Cohen’s dz (pairwise).

## 3. Results

This chapter presents findings and depletion rates from our series of battery tests described earlier. We also present results from our statistical analysis of the obtained results in order to judge the significance of the sensors’ impact on battery life using the previously presented methods.

### 3.1. Idle Test (RQ1)

**Figure 3:**
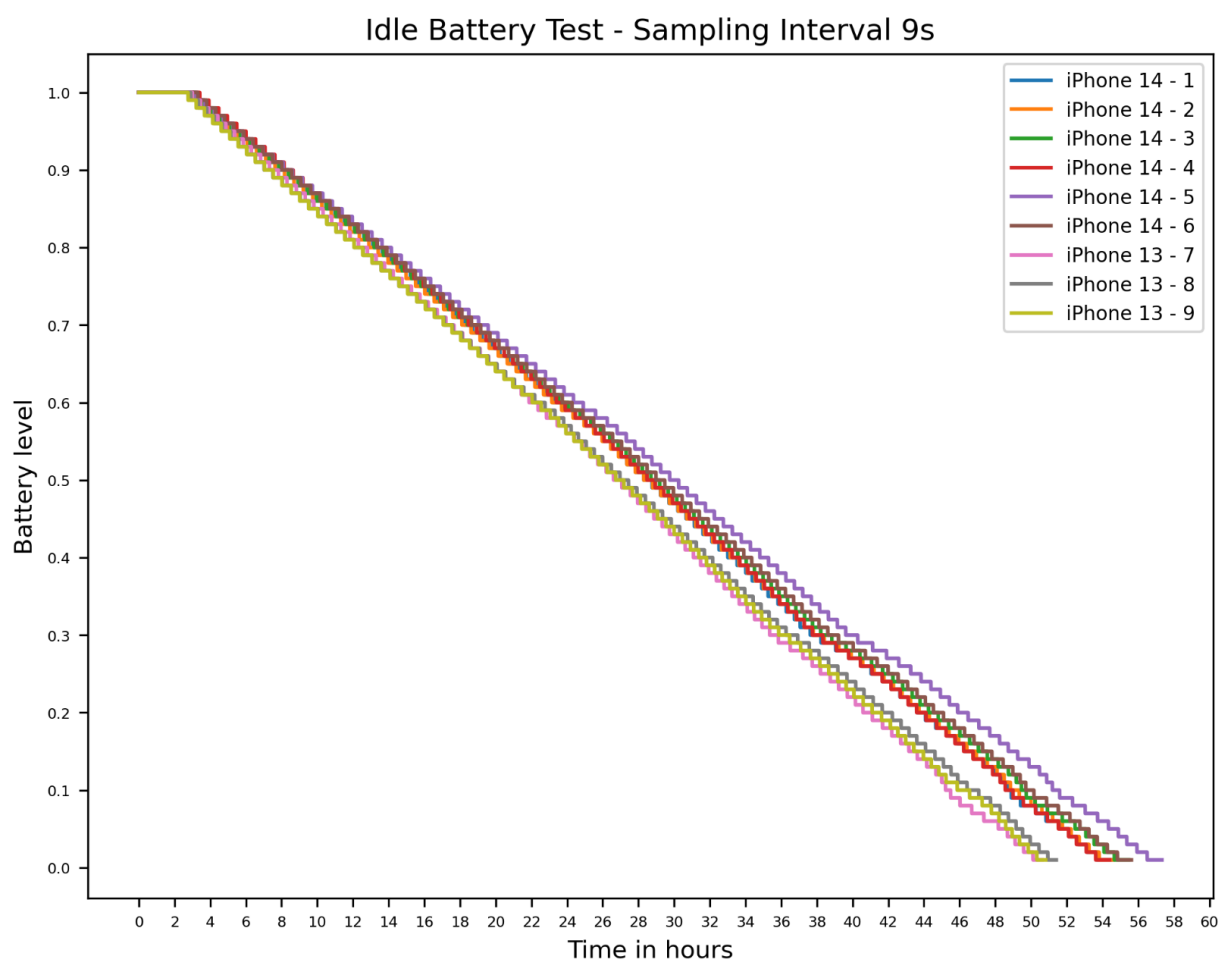
Idle test for iPhone 13 Pro and 14 Pro.

This section presents the baseline battery consumption needed of the devices that we measured to answer RQ1 through our idle test setup. Figure 3 shows the outcomes of the 9-s interval idle tests. As visible in the graph, the SoC decline over much of the run is approximately linear in our recorded traces. This is notable for the comparative analysis for the different sensors later. All iPhone 13 models had shorter battery life than the iPhone 14 models during this idle test. As the iPhone 14 has a battery with higher capacity, this result aligns with our expectations. The variation in run times was greater in the iPhone 14 models, spanning 2:55 h, compared to just 48 min for the iPhone 13 models. This leads to a coefficient of variation (CV) of 2.0% for the iPhone 14 models, and 0.8% for the iPhone 13 models. Hence, a greater variability in battery performance was observed in iPhone 14 models compared to iPhone 13 models.

**Figure 4:**
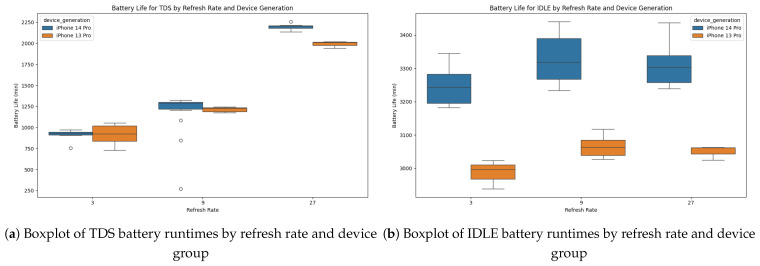
Boxplots of battery runtimes by refresh rate and device group: (**a**) TDS battery runtimes. (**b**) IDLE battery runtimes.

Next, we looked at the impact of different sampling rates on battery life during the idle tests. Judging by the median battery life of all iPhone 14 models, Figure 4 shows the impact of sampling rates. Interestingly, going from a 9-s to a 3-s sampling rate had a significant discrepancy in battery life, amounting to a CV of 1.7% for both iPhone families. Comparingly, we only observed disparities of up to 0.4% CV between the 27-s and 9-s sampling rates. A one-way repeated-measures ANOVA revealed a significant main effect of sampling rate on battery life during the idle tests, F(2,16)=88.43, p<0.001, partial $\eta^{2}=0.058$. Mauchly’s test indicated that the assumption of sphericity was met (W=0.945, p=0.822), so the uncorrected *p*-value is reported. Post hoc pairwise comparisons with Holm correction showed that battery life was significantly longer at both 9 Hz and 27 Hz compared to 3 Hz (3 vs. 9 s: t(8)=−11.31, p<0.001, $d_{z}=-0.534$; 3 vs. 27 s: t(8)=−10.35, p<0.001, $d_{z}=-0.448$). However, there was no significant difference between 9 Hz and 27 Hz (t(8)=2.08, p=0.071, $d_{z}=0.079$). We only carried out this analysis for the idle tests to get a general understanding of the overhead our software has on battery life based on the sampling rate.

### 3.2. TrueDepth Sensor Test (RQ1, RQ2)

Similarly to the idle tests, the iPhone 14 models showed an approximately linear SoC decrease over most of the TrueDepth test duration. Interestingly, the iPhone 13 models battery consumption was lower compared to the iPhone 14s up to the 20% mark. After that point, their consumption increased.

Excluding an iPhone 14 outlier with technical issues during the tests, the variability in run time for the iPhone 14 models was 33 min, compared to just 9 min for the other device group. The iPhone 14 models had a CV of 1%, more than double the 0.4% for the iPhone 13 models.

The different sampling rates had a more prominent impact on the devices’ battery life, as highlighted in Table 3. The difference in runtimes for the 3- and 9-s sampling rates varies by 10% for the iPhone 13 models and by 16% for the iPhone 14 models. Additionally, the difference from the 9-s to the 27-s sampling rate is approximately 40%. This trend of increasing runtime differences aligns with the tripling of the sampling rates from 3 to 9 to 27 s.

Paired two-tailed *t*-tests (Holm-adjusted, α=0.05, see Table 4) comparing each device’s idle run with its TrueDepth run showed that the sensor drains the battery significantly faster at every sampling interval we tested.

These very large effect sizes confirm the raw runtime losses from Table 3.

Additionally to the battery usage, we also analyzed the ratio of sensor data points to data points with just a timestamp and battery state. Ideally, this should be a one-to-one ratio, indicating that the sensor consistently delivered a value with respect to the sampling rate. This holds true for the iPhone 14 models with 9- and 27-s rates. The iPhone 13 models showcased a slightly below expected rate of about 46.5%, indicating that the sensor failed to deliver a value in reasonable time sometimes. For the test runs with 3-s rates, the observed ratios varied from 0.11% to 4.03%, highlighting major issues in rapid sensor usage during these tests. This issue will be explored in more detail in Section 4.

### 3.3. GPS Test (RQ1, RQ2)

Looking at the test runs with the highest location accuracy, the iPhone 14 Pro models showed a significantly longer battery life. Interestingly, while the SoC traces are approximately linear up to around 30% in our data, several deviations occur after this point. The range of runtimes on the iPhone 14 models was also notably large, extending over 6 h with a CV of 4.9%. Similarly, the iPhone 13 models showed a range of 2:19 h, resulting in a CV of 3.9%.

The GPS sensor was tested with each of the four location accuracy settings (10 m, 100 m, best, navigation) regarding significancy individually through *t*-tests comparing each test with the idle test on a given device.

Looking at the logged signals, a ratio of 23.5% to 36.1% for iPhone 13 models, and 29.9% to 42.87% for the iPhone 14 models were calculated. Interestingly, the GPS test with the lowest location accuracy setting yielded similar runtime results for both iPhone generations. The variance in this configuration was low, being 3 h for iPhone 14 models and 1 h for iPhone 13 models (CV of 2.1% for iPhone 14 models, and 1.0% for iPhone 13 models). Comparatively, the variance for the test with the highest location accuracy led to 6 h and 2 h, respectively. The significant difference in runtimes between the models was not observed with the 100 m accuracy setting. This setting also led to significantly lower battery consumption, with up to 20 h of additional runtime compared to the other configurations in iPhone 13 models.

### 3.4. Accelerometer, Gyroscope, and Pedometer Test (RQ1, RQ2)

The results for the Accelerometer, Gyroscope, and Pedometer tests exhibited similar results to the Idle tests with respect to both runtimes and graph patterns. During the Accelerometer test, the iPhone 13 models consistently showed shorter battery life, likely due to their smaller battery capacity. The iPhone 14 group had an outlier with consistently lower battery life and another outlier with above average performance, leading to high variability in Accelerometer test runtimes, ranging 5:37 h (CV of 3.4% for iPhone 14, and 0.6% for iPhone 13 Pro models).

**Figure 5:**
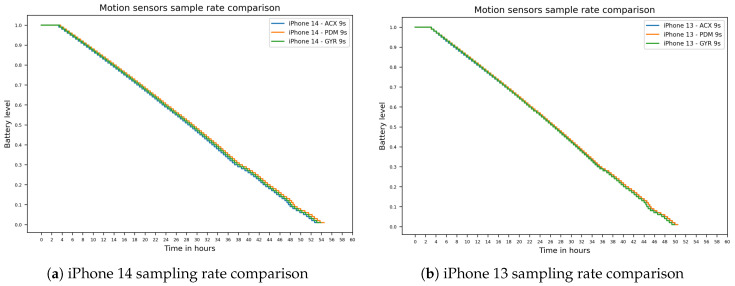
Runtimes for motion sensor tests: (**a**) iPhone 14 sampling rate comparison. (**b**) iPhone 13 sampling rate comparison.

The analysis of the sampling rates impact for the motion sensors lead to only marginal differences between the 27-s and the 9-s intervals, with a more pronounced difference between the 9-s and 3-s rates. While the trends are similar for all three motion sensors, it is least pronounced in the Pedometer tests. Looking at the highest sampling rate, the plots began to significantly deviate after the 55% mark, which is earlier than in the Idle tests. Figure 5 visually highlights the similar runtimes for all motion sensors at the 9-s sampling rate.

**Figure 6:**
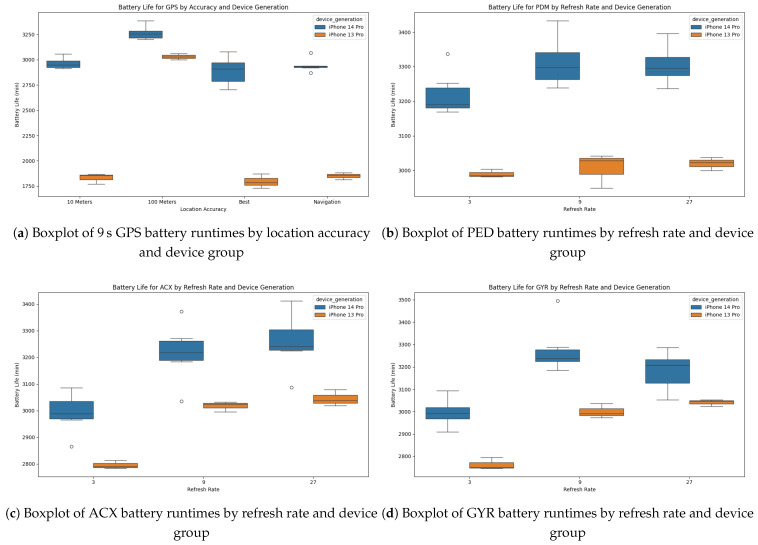
Boxplots of battery runtimes by configuration and device group: (**a**) GPS battery runtimes for the 9 s test, grouped by location accuracy. (**b**) PED battery runtimes by refresh rate. (**c**) ACX battery runtimes by refresh rate. (**d**) GYR battery runtimes by refresh rate.

The three motion sensors were tested regarding significancy individually through *t*-tests comparing each test with the idle test on a given device. The *t*-test results can be found in Table 2. We assume that these mostly insignificant results are at least partially based on the only marginal impact on battery life through the motion sensor tests compared to the other sensor tests. The corresponding boxplots in Figure 6b–d summarize the refresh-rate distributions for the Pedometer, Accelerometer, and Gyroscope tests, while Figure 6a provides the analogous distributional view for the GPS location-accuracy comparison discussed above.

Observing the logged value ratios, we noticed that the Pedometer did not register any step updates during our (static) test.

### 3.5. Camera Test (RQ1, RQ2)

**Figure 7:**
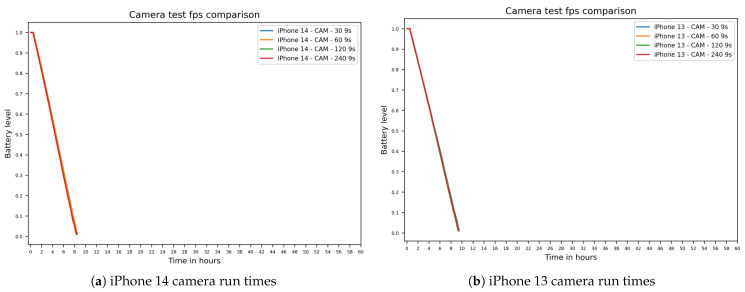
Runtimes for camera tests: (**a**) iPhone 14 camera run times. (**b**) iPhone 13 camera run times.

**Figure 8:**
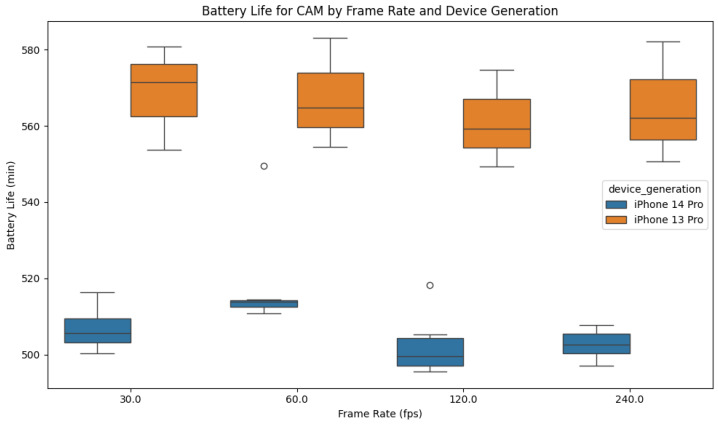
Boxplot of 9-s CAM battery runtimes by frame rate and device group.

The camera test exhibited a similar battery depletion to the TrueDepth sensor, but much steeper. This marks the camera as the sensor with the highest impact on battery life in our tests. Unexpectedly, the iPhone 13 models consistently showed lower depletion rates than the iPhone 14 models, leading to a median runtime difference of about an hour in favor of the older device generation. The different camera frame rates showed a negligible difference in runtimes, as seen in Figure 7 and Table 5. For instance, the 120 fps camera test resulted in shorter runtimes compared to the lower 30 fps setting, but the 240 fps setting unexpectedly showed longer runtimes than the 120 fps setting. The corresponding 9-s camera runtime distributions by frame rate and device group are summarized in Figure 8.

### 3.6. Cross-Sensor and Cross-Device Consistency (RQ2, RQ3)

**Figure 9:**
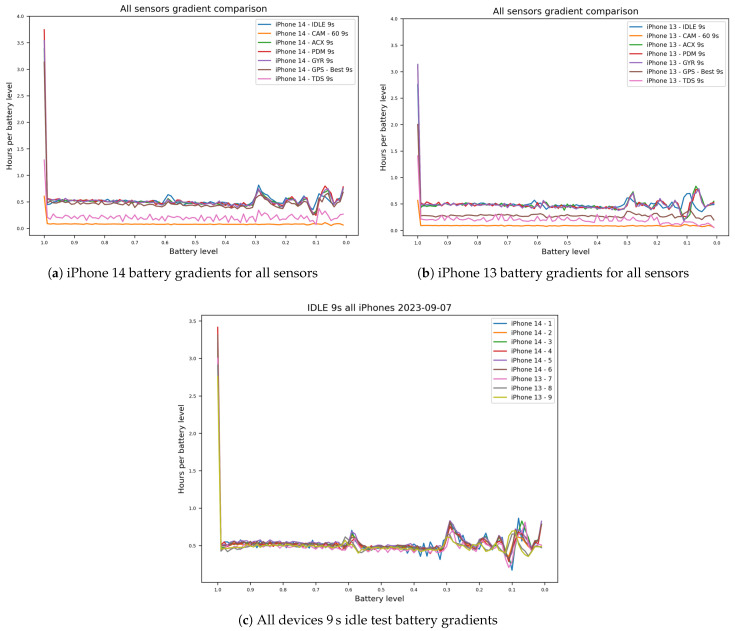
Battery gradients for mixed scenarios: (**a**) iPhone 14 battery gradients for all sensors. (**b**) iPhone 13 battery gradients for all sensors. (**c**) Battery gradients observed during the 9 s idle test across all devices.

This section analyzes battery depletion by examining the shape of the recorded SoC traces. These observations are empirical for our API-reported SoC data under the tested conditions and are not intended as a general claim about the electrochemical discharge curve of lithium-ion cells. This represents our approach to RQ3, comparing the iPhone 13 Pro and 14 Pro models across all scenarios. For this, we plotted the duration a phone kept a certain battery state against the battery state (see Figure 9).

Interestingly, the devices all exhibit prolonged duration of the full 100% charge state. Aside from this phenomenon, all devices fluctuate around the 0.5 h per battery charge percent mark. Slower decharge rates, as visible by the peaks in Figure 9, are visible at around 59%, 29%, and 9%. Aligning with previous results, the depletion rates below 20% diverge strongly.

For the GPS tests, we observed about 30 min per percent for the iPhone 14 models and 20 min for the iPhone 13 models, respectively. We could not find a clear depletion pattern for the TrueDepth test, indicating inconsistent power consumption by the sensor. As the depletion rate for the camera tests is significantly greater compared to the other tests (about 6 min per percentage), we switched to a reduced scale factor for Figure 9. This uncovered similar peaks to the other tests, indicating that they are proportionate to the depletion rate. The steep decline around the 29% mark in the idle test is not visible in the camera test results.

### 3.7. Battery Capacity Analysis

As some devices were already used in previous studies, they already started at an inconsistent cycle count. Additionally, due to the design of this study, the devices had different cycle counts for each sensor test. Due to these circumstances, we conducted an analysis on the impact of the cycle count on battery capacity and battery life.

**Figure 10:**
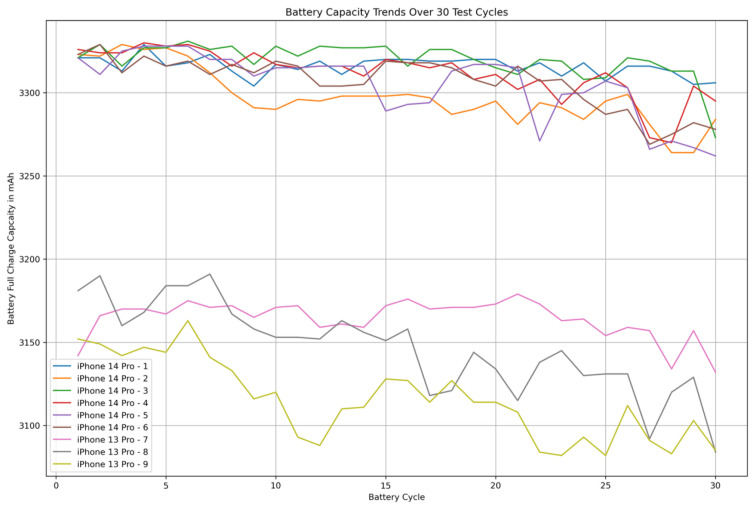
Battery capacity trends for all devices.

Figure 10 clearly shows clustering of the iPhone 14 devices and the iPhone 13 devices throughout the tests. The initial battery capacity of the devices varied slightly, ranging from 3321 mAh to 3326 mAh for the iPhone 14 models and 3142 mAh to 3181 mAh for the iPhone 13 models. While the capacity changes for the first five test cycles in the 14 Pro models was negligible, it increased in later cycles. Over the 30 cycles, a general downward trend for battery capacity for the 14 Pro models was observed. As the iPhone 13 models were not new and already had charging cycles, they had a larger absolute and relative difference in battery depletion compared the 14 Pro models. Notably, the pre-used iPhone 13 Pro devices showed larger fluctuations in battery capacity, with an up to 3.05% decrease in capacity.

We also conducted a comparative analysis of two idle tests with identical configurations to determine the effect of the reduced battery capacities. These were test cycles 4 and 26. The relative runtime differences between these tests ranged from −0.94% to 0.48% (CV of −0.51%).

## 4. Discussion

### 4.1. Study Results

We used the coconutBattery software to monitor the devices’ charging state. This was critical, as we observed incongruities regarding the devices’ built-in software. Specifically, we found that it lacked the necessary precision towards the 100% charging state. The devices often already showed a 100% state, even though the precise measurement by coconutBattery was between 95% and 100%. This suggests that the iPhone’s battery indicator is not representative of the real physical battery state and rather a software layer meant to display an understandable representation of charging state to the user. Figure 9 supports this assumption by showing the majorly extended duration of devices at 100% battery state during our tests. Notably though, using coconutBattery also came with issues. We observed that we could reliably charge devices another ∼15 mAh even after the software showed a 100% battery state. Given these issues, we decided to deem a device “fully charged” once coconutBattery showed 100%. These minor inconsistencies could explain some of the fluctuating battery seen in Figure 10.

By using 6 iPhone 14 Pro devices and only 3 iPhone 13 Pro devices, we might have introduced a potential bias towards higher variability in the iPhone 14 group’s battery performance and run times, as the larger group naturally increases the likelihood of variance in battery capacity. As such, comparisons regarding battery variance between the groups should be studied further before drawing final conclusions. To increase interpretability of cross-generation comparisons (RQ3), we recorded each device’s maximum battery capacity before runs (see Figure 10) and considered these values when interpreting group differences.

We noticed issues with varying light levels in our preliminary tests, which we stabilized by adding a powerbank-powered LED light source in the box. This is relevant regarding the reproducibility of our results—a darker environment might lead to issues with the TrueDepth sensor failing to activate in time, depending on the sampling rate. All our devices were unable to recognize the printed face after the respective battery state dropped below 20%. We believe that the “low battery” system alert prevented the sensor from being activated while displayed, which is consistent with previous internal research. Surprisingly, the battery depletion rates of the iPhone 13 devices increased after reaching 20%. The iPhone 14 Pro models did not encounter this issue. This is likely due to the difference in how the “low battery” alert is displayed on these devices. Instead of a system alert that covers the currently running application’s UI, a small indicator is shown in the dynamic island at the top of the screen. The results of the 3-s sampling rate TrueDepth tests might also include limitations, as we understand that the sensors rarely managed to activate during the 3-s interval. This sampling rate might have been too quick, especially considering the face being printed and the lighting being suboptimal. As expected, given the large battery life disparities, all three sampling rates produced statistically significant results.

As we implemented a static test setup with a box inside a building, only using the GPS sensor was not sufficient, forcing us to enable WiFi to improve accuracy. Importantly, enabling this second hardware sensor adds an unquantified power component. Therefore, we did not isolate and calculate true GPS-only energy consumption in this setup; the reported GPS results should be interpreted as GPS+WiFi under controlled indoor conditions. However, telling users to enable WiFi for improved location accuracy is standard practice in many consumer applications [37]. In addition to the usage of the WiFi sensor, the indoor environment might have also influenced the GPS sensor itself. A dynamic outdoor environment might lead to a higher hardware sampling rate (a factor that cannot be controlled through the CoreLocation API) and thus increased battery consumption. Through separate internal tests, we observed this to be as frequent as every second (compared to the 12 s in stationary tests) in a small separate test setup. All GPS sensor configurations were shown to have a statistically significant impact on battery life, as demonstrated by the paired two-tailed *t*-test results we presented in the previous section. When comparing device groups, we noticed that in high-accuracy scenarios, the iPhone 13 models significantly underperformed the newer device group. This did not occur for the lower accuracies. As we conducted our tests under identical OS and software versions, this behavior is likely linked to a difference in hardware components. Notably, the iPhone 14 Pro is equipped with a dual-frequency GPS chip [38] compared to the single-frequency GPS chip in the iPhone 13 Pro [39]. Interestingly, Apple recommends using the best for navigation accuracy only while the device is charging [40]. Contrary to that, we observed slightly longer run times for both device models with this configuration. This finding suggests that the “best for navigation” mode involves an approach that draws more battery while the device is in motion.

Despite our static test setup, we observed that both the accelerometer and the gyroscope successfully collected data, including detection of gravity. As the devices were not moved, the pedometer did not collect any step data. This reflects a deliberate study design choice: to estimate baseline costs of activating these sensors in a standardized, low-variance laboratory environment. Accordingly, these values should be interpreted as controlled baseline estimates rather than direct representation of real-world movement-heavy usage. This static setup might have impacted the sensors operation, leading to non-representative battery usage. This would make sense given the slightly longer median runtime we observed in the pedometer tests. The difference was minimal though, falling within the CV across different devices and with mostly statistically insignificant results. As such, we cannot be sure about the impact of the test setup on the pedometer battery impact, necessitating further research in this direction.

Unexpectedly, we found similar results across different camera configurations, which might be caused by the sensor test software we developed. To be able to select different frame rates in the battery test app, our software selects the first of potentially many camera configurations returned by AVFoundation [41] that includes this frame rate. This could have led to inconsistencies regarding resolution or other settings, leading to the uniformity of our data. For example, the higher frame rates (120 and 240) only support 1080p resolution, while the lower frame rates (30 and 60) can go up to 4k. The sensor test app does not render a live camera preview and does not store recordings, by design, as this protocol aims to isolate baseline camera-activation cost while minimizing additional computational and storage overhead. This camera configuration is consistent with the rest of our protocol, where sensor outputs are generally not rendered on-screen during measurements. Even under this reduced-load setup, camera conditions still showed substantially reduced battery life and statistically significant effects, including the camera and GPS test, supporting practical relevance as a lower-bound estimate of camera-related battery cost. At the same time, real-world camera use typically includes preview rendering, encoding, and storage, so those scenarios can be expected to draw additional power beyond the baseline measured here. All of these issues imply the need for more detailed exploration of this sensor and its impact on battery life.

### 4.2. Research Questions

#### 4.2.1. Answer to RQ1

We established the upper bound of battery life as 36:44 h on the 14 Pro models and 33:30 h on the 13 Pro models for the 27 s idle tests using our proposed standardized protocol. High-accuracy GPS shortened the endurance roughly five-fold, resulting in ∼30 h, and 20 h for the new and old devices, respectively. The TrueDepth tests ran for 36:44 h on the new devices and 33:30 h on the 13 Pro models. The three motion sensors showed similar battery performance and respective graphs, with about 54 h on the 14 Pro models and about 50:30 h on the 13 Pro models. Camera, GPS, and TrueDepth tests lead to statistically significant results, with the motion sensor tests producing mixed results. These results are summarized by the following hierarchy in descending order of median battery life for the iPhone 14 Pro group.

1. Idle
2. Pedometer
3. Accelerometer
4. Gyroscope
5. TrueDepth
6. GPS
7. Camera

#### 4.2.2. Answer to RQ2

As we presented in Section 3, increasing the sampling rate from 27 s to 9 and 3 s already showed an impact on battery life. This difference is more pronounced in the sensor tests, with the TrueDepth showing 57% reduced runtime with the fastest sampling rate compared to the slowest sampling rate. While the trend remains in place for the motion sensors, it is not as pronounced as seen in Table 6. We were not able to measure any impact of camera frame rate on battery life during our tests, which we attribute to software issues. Choosing lower location accuracies led to significantly increased battery life in our GPS tests, showcasing that developers have a tradeoff between accuracy and cost.

#### 4.2.3. Answer to RQ3

Battery impact was not lower on the newer device generation across all sensors, with differences between generations depending on the sensor in use. During the idle test, the 14 Pro models outlasted the 13 Pro models by about 10%. A notable difference was found during the GPS tests with high accuracy, as the 14 Pro models lasted about 30 min per 1% of charge, compared to just 20 min on the 13 Pro models, leading to a 50% lead in battery life. During the TrueDepth tests, the groups behaved similarly up to the 20% mark, after which they converged. Most interestingly, we observed opposite behavior in the camera tests. The older device group lasted about an hour longer, suggesting that there might have been changes in camera software and hardware that led to increased energy usage across device generations. Motion sensors behaved similarly to the idle tests, with only mild differences across groups. In total, these results showcase that cross-generation consistency is heterogeneous. This underscores the need for sensor- and device-specific benchmarking using a standardized protocol when assessing battery life.

### 4.3. Comparison to Existing Work

The literature discussed in the introduction suggests three complementary perspectives on mobile energy measurement: invasive hardware instrumentation, software- or model-based profiling, and application-level sensor benchmarking. Our work most directly belongs to the third category, but it is positioned relative to the other two by intentionally trading some measurement granularity for lower setup cost and easier reproducibility on contemporary iPhones. In their 2009 study on energy-efficient mobile sensing solutions, Abdesslem et al. [24] compared the battery life of camera, GPS (indoors and outdoors), microphone, Bluetooth, accelerometer, and an idle setup similar to ours on a Nokia N95 device. While their goal was somewhat different to ours, as they tried to improve battery performance while sensing, the results are interesting. They found that the accelerometer consumed the least energy, which aligns nicely with our motion sensor tests. Interestingly, they found that the camera had about 30% the battery life compared to an indoor GPS test, which is not the case in our observations. Of course, this is likely due to the advancements in hardware in the past 16 years. Another interesting finding is that the aforementioned accelerometer battery life was only about 27% that of the idle test, which also is not comparable to our results. This probably aligns with more complex operating systems since then. Our results align well with the finding by Katevas et al., whose observation that GPS with best location accuracy draws the most power we could confirm despite studying devices that are over 10 years newer [22]. Compared to software-based profilers such as PowerTutor, DevScope, or Eprof, our protocol provides less fine-grained attribution to individual processes or hardware states, but it requires neither device-specific power-model training nor invasive modification of the smartphone hardware [18,19,20]. Compared to hardware-based approaches such as Carroll et al. or GreenMiner, the presented protocol is simpler to employ and easier to reproduce in regular lab workflows, at the cost of relying on software-visible battery state and end-to-end runtime instead of direct electrical measurements [15,16].

### 4.4. Limitations and Threats to Validity

During the first test run, a person visiting the lab opened and moved the device box without permission, which led to unusable data. Given this issue, a future protocol should include instructions for outside people on how not to interact with the setup. Additionally, we recommend locking the device box so it can only be opened by authorized personnel. As the devices are locked in a wooden box inside a solid building, some sensors might experience out-of-the-ordinary circumstances. The GPS sensor, for example, could be subjected to drift, as the GPS satellite connection is flawed in the setup. As such, it might behave out of the norm, with potentially more update requests than if the connection were stable and if there was no drift. It is in the nature of this lab experiment test setup that it is not a realistic usage scenario for some sensors. The enclosed setup was intentionally chosen to isolate sensor-specific effects under controlled conditions, but this same isolation limits ecological validity. Moreover, our environmental control was based on a standardized ordinary indoor workspace (same building location, closed windows, low people traffic, and frequent overnight runs) rather than a dedicated environmental chamber, so room-level influences were reduced but not fully eliminated or visually documented beyond the box photograph. The choice for a regular office space instead of a dedicated chamber is also based on the goal of presenting a low-cost and reproducible protocol. For example, many real-world applications (such as navigation) are used outdoors with increased device temperature, high ambient light and often near-maximum display brightness, which can substantially increase power draw relative to our dark-box protocol. Likewise, real-world sensing commonly combines multiple active sensors rather than the mostly isolated conditions used here. Given this, it is advisable to conduct similar battery tests on a realistic, but controlled, scenario as well. For example, the same software setup could be used on the go for realistic data collection. Sample size (both device count and number of independent runs per exact configuration) was limited for this initial study, so future work should expand on this. More specifically, our evidence is strongest for the repeatedly measured baseline and key comparison conditions (e.g., idle 9 s, TrueDepth 9 s, and the repeated GPS configurations), whereas several camera and motion-sensor configurations were executed only once and therefore provide lower-confidence estimates of absolute runtime. We partially addressed this limitation by running all devices under the same protocol, reporting medians and coefficients of variation, and restricting formal repeated-measures inference to conditions with matched observations across devices. Additionally, due to the closed nature of iOS, background and system-level processes cannot be fully controlled and may still influence battery depletion despite our standardized software setup. In addition, our battery analyses rely on software-reported SoC during runs (via the iOS `UIDevice’ battery API); while pre-test charging was cross-checked with coconutBattery, we did not employ hardware-level power instrumentation to adhere to our low-cost and reproducible protocol requirements. Relatedly, we did not bypass the internal battery with an external power supply, so battery voltage dynamics during discharge cannot be fully eliminated as a potential influence. To mitigate this effect within the chosen low-cost design, we standardized the pre-test charging state at a verified 100% SoC, applied identical software and environmental preparation steps, and compared conditions using repeated runs with median-based summaries under the same protocol.

### 4.5. Future Work

We propose to use the presented sensor battery test protocol to validate changes in hardware and software of newer devices. Examining the sensors that exhibited unexpected or uncontrolled behavior in our suite of tests would be recommended. In particular, we believe that comparing different approaches to ensuring and verifying the intended 100% battery state before each test run (other than the system indicator and coconutBattery, the third-party software we used) could further increase the reliability of our results. Future sensor tests should include sufficient device group sizes, in particular, based on our variability in the smaller iPhone 13 Pro group. Future work should explicitly model and control battery degradation effects in more detail, ideally combining larger samples with hardware-based battery measurements. Other device types and devices from other manufacturers should also be tested for sensor battery impact. Future protocol iterations should also include continuous in-test temperature monitoring (rather than only pre-run checks) to quantify thermal drift during long experiments and its potential influence on battery depletion. Doing foundational research into face recognition and performance implications of real face vs. mannequin vs. printed face for the TrueDepth sensor could provide interesting insights to refine the protocol for increased real-world reliability. The GPS and motion sensors should also be tested in more dynamic, real-world use cases, requiring an adapted protocol that still fulfills our requirements while producing more statistically significant results. In particular, the pedometer needs to be tested in a real-world scenario, as we assume that it was likely inactive during our tests. Future studies should explicitly include outdoor conditions to quantify how much environmental exposure alters the sensor-specific battery estimates obtained from our laboratory protocol. In addition, future studies should include configurations for display brightness to quantify its impact on battery life. Developing more rigid software for future camera tests would be advisable. This entails configuration of frame rate and resolution to ensure consistent setups. The camera should also be tested while displaying the image feed on the screen, as this might lead to an increase in computational load, resulting in increased battery drainage. With iOS 18, Apple restricted access to the battery level through the on-device API we used in the BatteryTest application. In particular, developers now only have access to rounded (to the closest 5 percent) battery values. This implies a need to adapt the analysis of test results, as detailed battery level graphs and gradients are not reproducible anymore. Total battery life per sensor can still be measured as presented in this paper. Alternative approaches could also be based on adapted battery state measuring, such as dedicated hardware reading charge rates. Despite this unfortunate change, the presented protocol is still recommended for future device sensor battery impact tests.

## 5. Conclusions

We proposed a reproducible, low-cost protocol that combines strict hardware preparation, a unified software checklist, and the custom BatteryTest app to measure the energy cost of individual iPhone sensors. Thirty independent analyzed runs on 9 iPhone 13 Pro and iPhone 14 Pro devices showed a clear hierarchy of power demand: the rear camera was the most energy-intensive, high-accuracy GPS and TrueDepth sat in the middle, and inertial sensors hovered close to idle drain. Sampling rate mattered, as cutting the interval from 27 s to 3 s raised consumption by up to 40%—and hardware generation effects were sensor-specific, with the 14 Pro saving power in GPS but draining faster during camera use.

The lab setting necessarily omits motion and outdoor reception, and small discrepancies in charge verification and cohort size limit generalization. Future work will extend the protocol to dynamic, real-world scenarios, refine camera tests to separate frame rate from camera resolution, and adopt higher-precision charging checks. Even in its current form, the protocol offers developers and researchers a practical foundation for energy-aware design and longitudinal benchmarking of new smartphone hardware. Our insights enable developers to judge which sensors should be used for everyday applications and which battery life tradeoffs they should consider.

## Figures and Tables

**Table 1:** System configuration settings applied during all experiments.

Setting	State
Light Mode	On
Do Not Disturb	On
Airplane Mode	On
Auto-Lock	Off
Automatic Light/Dark Appearance	Off
Screen Time	Off
Location Services	Off
Find My iPhone	Off
Exposure Notifications	Off
Automatic Updates	Off
Wi-Fi	Off
Bluetooth	Off
Background App Refresh	Off
Auto-Brightness	Off
Optimized Battery Charging	Off

**Table 2:** Overview of independent analyzed runs per scenario.

Scenario	Independent Runs	Configurations
Idle	5	3 s, 27 s, 9 s, 9 s, 9 s
TrueDepth	6	3 s, 9 s, 9 s, 27 s, 3 s, 9 s
GPS	5	100 m, 10 m, 100 m, best, best
Accelerometer	3	3 s, 27 s, 9 s
Pedometer	3	3 s, 27 s, 9 s
Gyroscope	3	27 s, 3 s, 9 s
Camera	5	30 fps, 60 fps, 120 fps, 240 fps, 60 fps with GPS

**Table 3:** Comparison of median performance times and percentages for TDS test.

	TDS 27 s	TDS 9 s	TDS 3 s
Median 14 Pro	36:44:20	21:40:48	15:48:20
Median 13 Pro	33:30:08	20:36:45	17:03:05
Median 14 Pro %	100.0%	59.0%	43.0%
Median 13 Pro %	100.0%	61.5%	50.9%

**Table 4:** *T*-test results for all hardware sensors and sampling rates.

Configuration	t-Value	*p*-adj	d-z	Significant
GPS 10 m	4.45	0.0171	1.48	yes
GPS 100 m	7.12	0.0011	2.37	yes
GPS best	4.96	0.011	1.65	yes
GPS navigation	4.71	0.0136	1.57	yes
TDS (27 s)	76.2	n/a	25.4	yes
TDS (9 s)	33.8	n/a	11.3	yes
TDS (3 s)	50.0	n/a	16.7	yes
ACX (27 s)	1.52	0.501	0.51	no
ACX (9 s)	2.82	0.136	0.94	no
ACX (3 s)	9.32	0.000172	3.11	yes
GYR (27 s)	2.18	0.304	0.73	no
GYR (9 s)	2.93	0.132	0.98	no
GYR (3 s)	28.98	n/a	9.66	yes
PED (27 s)	1.42	0.501	0.47	no
PED (9 s)	2.14	0.304	0.71	no
PED (3 s)	1.39	0.501	0.46	no
CAM (30 fps)	48.29	n/a	16.10	yes
CAM (60 fps)	49.16	n/a	16.39	yes
CAM (120 fps)	48.46	n/a	16.15	yes
CAM (240 fps)	48.09	n/a	16.03	yes
CAM + GPS	60.21	n/a	20.07	yes

**Table 5:** Comparison of median performance times for camera configurations.

Device	30 FPS 9 s	60 FPS 9 s	120 FPS 9 s	240 FPS 9 s
iPhone 14 Pros	08:25:42	08:33:53	08:19:34	08:22:38
iPhone 13 Pros	09:31:30	09:24:44	09:19:20	09:22:12

**Table 6:** Overview of median runtimes for Idle, TDS, ACX, GYR, and PDM tests.

Test	27 s	9 s	3 s
Idle (14 Pro)	55:03:52	55:01:32	54:02:55
Idle (13 Pro)	51:01:45	50:48:53	49:56:29
TDS (14 Pro)	36:44:20	21:40:48	15:48:20
TDS (13 Pro)	33:30:08	20:36:45	17:03:05
ACX (14 Pro)	54:01:24	53:37:59	49:48:53
ACX (13 Pro)	50:38:26	50:24:23	46:29:22
GYR (14 Pro)	53:26:57	53:57:39	49:52:55
GYR (13 Pro)	50:46:29	49:50:49	45:49:58
PDM (14 Pro)	54:56:02	54:59:05	53:10:42
PDM (13 Pro)	50:23:05	50:28:10	49:45:02

## Data Availability

The corresponding data is available at the Bonndata data repository under DOI: 10.60507/FK2/LSJGWW [33].
